# Variability in estimated glomerular filtration rate values is a risk factor in chronic kidney disease progression among patients with diabetes

**DOI:** 10.1186/s12882-015-0025-5

**Published:** 2015-03-25

**Authors:** Chin-Lin Tseng, Jean-Philippe Lafrance, Shou-En Lu, Orysya Soroka, Donald R Miller, Miriam Maney, Leonard M Pogach

**Affiliations:** Department of Veteran Affairs-New Jersey Health Care System, 385 Tremont Avenue, Mail Stop#15, East Orange, NJ 07018 USA; Department of Preventive Medicine and Community Health, Rutgers University, New Jersey Medical School, Newark, NJ USA; Department of Medicine, University of Montreal, Montreal, QC Canada; Department of Biostatistics, Rutgers School of Public Health, Piscataway, NJ USA; Bedford VA Medical Center, Center for Health Quality, Outcomes and Economic Research, Bedford, MA USA; Boston University, School of Public Health, Boston, MA USA

**Keywords:** Chronic kidney disease, Diabetes, Dialysis, Glomerular filtration rate, Mortality

## Abstract

**Background:**

It is unknown whether variability of estimated Glomerular Filtration Rate (eGFR) is a risk factor for dialysis or death in patients with chronic kidney disease (CKD). This study aimed to evaluate variability of estimated Glomerular Filtration Rate (eGFR) as a risk factor for dialysis or death to facilitate optimum care among high risk patients.

**Methods:**

A longitudinal retrospective cohort study of 70,598 Veterans Health Administration veteran patients with diabetes and CKD (stage 3–4) in 2000 with up to 5 years of follow-up. VHA and Medicare files were linked to derive study variables. We used Cox proportional hazards models to evaluate association between time to initial dialysis/death and key independent variables: time-varying eGFR variability (measured by standard deviation (SD)) and eGFR means and slopes while adjusting for prior hospitalizations, and comorbidities.

**Results:**

There were 76.7% older than 65 years, 97.5% men, and 81.9% Whites. Patients were largely in early stage 3 (61.2%), followed by late stage 3 (28.9%), and stage 4 (9.9%); 29.1%, 46.8%, and 73.3%, respectively, died or had dialysis during the follow-up. eGFR SDs (median: 5.8, 5.1, and 4.0 ml/min/1.73 m^2^ ) and means (median: 54.1, 41.0, 27.2 ml/min/1.73 m^2^) from all two-year moving intervals decreased as CKD advanced; eGFR variability (relative to the mean) increased when CKD progressed (median coefficient of variation: 10.9, 12.8, and 15.4). Cox regressions revealed that one unit increase in a patient’s standard deviation of eGFRs from prior two years was significantly associated with about 7% increase in risk of dialysis/death in the current year, similarly in all three CKD stages. This was after adjusting for concurrent means and slopes of eGFRs, demographics, prior hospitalization, and comorbidities. For example, the hazard of dialysis/death increased by 7.2% (hazard ratio:1.072; 95% CI = 1.067, 1.080) in early stage 3.

**Conclusion:**

eGFR variability was independently associated with elevated risk of dialysis/death even after controlling for eGFR means and slopes.

**Electronic supplementary material:**

The online version of this article (doi:10.1186/s12882-015-0025-5) contains supplementary material, which is available to authorized users.

## Background

Chronic kidney disease (CKD) affects 15% of the U.S. adult population and is associated with a high burden of morbidities and mortality [1,2]. Patients with CKD are a heterogeneous group characterized by varying rates of progression to a devastating outcome such as end-stage renal disease (ESRD), likely due to presence/absence of various risk factors [3-6]. Identifying risk factors of CKD progression or death will directly benefit patients by helping clinicians optimize health care and provide proper referral to multi-disciplinary clinics based on patients’ risk profiles, hence may help reduce patients’ risk of morbidities and mortality. Existing literature has provided support for many risk factors of CKD progression or death. A greater degree of estimated glomerular filtration rate (eGFR) downward trend was found to be associated with higher mortality [7]. Other identified predictors of CKD progression included demographics (age, sex, race), laboratory test results (proteinuria, phosphorus, albumin, hemoglobin), and comorbidities (diabetes, vascular disease, and hypertension) [3,7,8]. Recently, an algorithm was proposed to combine those factors in predicting a patient’s progression [9]. However, CKD progression is usually not constant over time [10-12]. For example, acute kidney injury—sudden deterioration of kidney function, is a critical adverse event in CKD progression [13-15].

eGFR is often used in tracking CKD progression. Although it is well recognized by clinicians that a single eGFR may not provide sufficient and complete information to one’s kidney function. For instance, two patients with the same eGFR value may have different resilience to kidney offense. Namely, other risk factors being equal, an adverse event or an acute disease (i.e., a stimulus) such as an infection may deteriorate kidney function (less renal resilience) in one patient but not in the other (greater resilience). In this study, we have proposed to use variability of eGFR values to measure kidney resilience, with larger eGFR variability indicating reduced kidney resilience to stimuli, and considered the state of kidney resilience as a potential risk factor of CKD progression. There are studies that have attempted to use repeatedly measured eGFRs to model CKD progression using different statistical approaches, including modeling of non-linear trajectories [12,16-18]. A recent study measured variability of kidney function using repeated measured eGFRs [19] and found it to be associated with increased risk of death in stage 3–5 CKD patients.

In this study, we also aimed to evaluate eGFR variability as a risk factor; however, we have used different study designs and methods, and included not only death but also dialysis as the adverse CKD outcomes of interest. Our hypothesis is that greater eGFR variability is associated with a higher risk of incurring dialysis or dialysis-free death, independently of the CKD progression trend. In essence, we believe although renal function decline may be inevitable for patients with CKD, those with similar decline rate of eGFR may be further differentiated by degree of renal resilience, as reflected by eGFR variability.

We chose to focus on CKD patients with coexisting diabetes in the study. Patients with diabetes are at higher risk of developing CKD. The risk of morbidity and mortality increase in CKD patients with diabetes [20], highlighting the importance of assessing and managing risk factors associated with progression of CKD. Furthermore, both conditions have considerable costs and negative impacts to individuals, health care systems, as well as the society as a whole, and they are increasingly prevalent. In the Veteran Health Administration (VHA) health care system, about one in five veterans have diabetes [21] and 30% of veterans with diabetes have CKD [22,23].

## Methods

### Study design, data sources and study population

This was a longitudinal retrospective cohort study of VHA veteran patients with diabetes in fiscal year (FY) 2000 (9/30/1999-10/1/2000) from a national research database containing linked longitudinal patient records of inpatient and outpatient services [21]. We determine the baseline CKD status using two outpatient eGFR values less than 60 ml/min/1.73 m^2^: the first (index) eGFR value was identified in FY2000, followed by a subsequent qualifying eGFR 90 to 365 days apart from the index eGFR. [23] eGFR was calculated with the four-variable version of the Modification of Diet in Renal Disease equation [24,25]. The baseline period was the 12 months prior to (and including) the date of the qualifying eGFR. Individuals were followed through FY2004, with follow-up time ranging between less than a year and close to five years.

**Figure 1:**
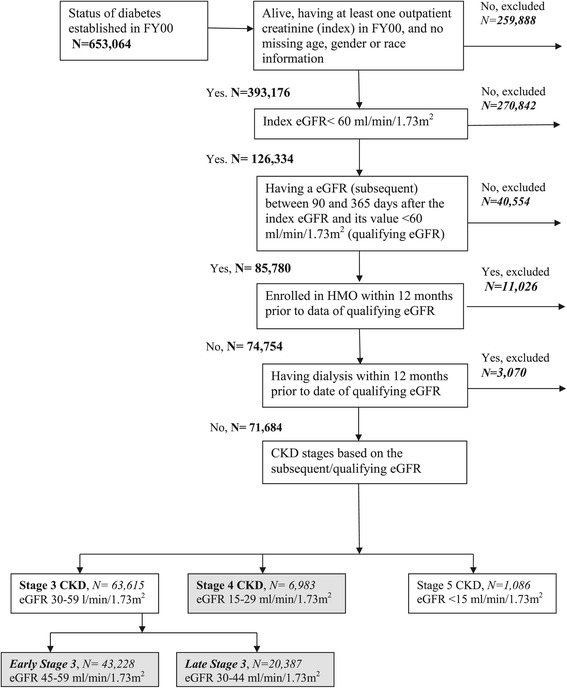
**Inclusion and exclusion steps for the creation of the study population, veteran patients with diabetes and chronic kidney disease stages 3 and 4, USA.** CKD – Chronic Kidney Disease; eGFR - estimated Glomerular Filtration Rate; FY – Fiscal Year (FY 2000: 10/1/1999-30/9/2000); HMO: Health Maintenance organization; USA – United States of America. Grey-shaded boxes indicate patient subgroups (by chronic kidney disease stage) included in the final study population.

Of the 653,064 VHA clinic users with diabetes in FY2000, we excluded individuals according to different criteria detailed in Figure 1. Patients enrolled in Medicare Health Maintenance Organization (HMO) were excluded because detailed Medicare HMO data is not routinely collected by Center for Medicare and Medicaid Services. We also excluded individuals with evidence of prior dialysis, using International Classification of Diseases, Ninth Edition, Clinical Modification (ICD-9-CM) diagnosis and procedure codes, Current Procedural Terminology codes, revenue codes (Medicare Part A), and VHA clinic stop codes ([23]; Additional file 1). We determined CKD stage using the qualifying eGFR based on the National Kidney Foundation Kidney Disease Outcome Quality Initiative (K/DOQI) criteria [24], and divided stage 3 into early (eGFR 45–59 ml/min/1.73 m^2^) and late stage (eGFR 30–44 ml/min/1.73 m^2^) [1,23]. We excluded individuals in stage 5 CKD, who are close to developing ESRD.

The VHA New Jersey Healthcare System Institutional Review Board approved the study. This study received a waiver of consent from the facility Internal Review Board.

### Outcome measures

The outcome was time from the date of the qualifying eGFR to the event of dialysis or dialysis-free death, whichever came first. The event time was censored if the individual was enrolled in Medicare HMO or alive at the end of FY 2004. Death information came from the VHA Vital Status File [26]. We ascertained dialysis using the method described above, but selected codes explicitly referring to dialysis treatment (see Additional file 1).

### Independent variables

The primary exposure variable was variability in the outpatient eGFR values. For each patient, we calculated standard deviations (SDs), concurrent means, and slopes of eGFR progression (derived from a linear regression) of repeatedly measured eGFRs in a series of (five) two-year moving intervals. In our analyses, the number of actual eGFR measurements per patient was at least two in each of the five intervals. These variables were later used as time-varying variables in Cox regression models. Other independent variables included demographic variables (age, sex, and race/ethnicity), VHA priority status, and clinical factors: prior hospitalization and medical comorbidities, all derived from the baseline period. The VHA categorizes veteran enrollees into 1 of 8 mutually exclusive priority groups [27] based on poverty level and service connected disability. We regrouped patients into four levels (severely disabled, moderately disabled, poverty, and co-payment required). We applied the Selim Comorbidity Index [28] to determine health status using ICD-9-CM codes, including indicators of physical (cardiovascular and non-cardiovascular conditions separately), and mental comorbidities. Cardiovascular conditions included angina pectoris, myocardial infarction, congestive heart failure (CHF), peripheral vascular diseases, and stroke. Mental comorbidities included schizophrenia, depression, bipolar disorder, anxiety, post-traumatic stress disorder, and alcohol abuse.

### Statistical analysis

We fitted the Cox proportional hazards models to evaluate the association between eGFR variability and risk of dialysis or dialysis-free death by each CKD stage. Prior to statistical modeling, we evaluated possible correlation/association among independent variables and found little indication of co-linearity among variables within each model.

Primary analysis: We divided the follow-up time into yearly intervals and included the eGFR SDs, means and slopes (derived from the two-year interval prior to each yearly interval in the follow-up) as lagged time-dependent covariates. Because higher values (hence greater means) are usually associated with greater SDs, and lower values (smaller means) are associated with smaller SDs, we included both SDs and means (derived from the same time intervals) as time-dependent covariates in the Cox models. We also included interactions of eGFR SDs and slopes to assess if the influence of eGFR variability differs with changes in the slopes.

Sensitivity analysis: We conducted four sensitivity analyses to evaluate robustness of the findings from the primary analysis. First, regarding the length of the time interval used to derive the time-varying eGFR SDs, means and slopes, we re-fitted Cox regression models using re-calculated time-varying eGFR SDs, means and slopes from a one-year interval (prior to each yearly interval in the follow-up). Second, we re-divided the follow-up time into quarterly intervals and included eGFR SDs, means and slopes from prior eight quarters (two years) as time-dependent variables in Cox regression models. Third, we examined the effect of extreme slopes on our main findings by removing those with absolute values greater than 36.5 or 25.0 ml/min/1.73 m^2^ per year; this also simultaneously removed extreme SDs. Fourth, as an alternative, for each patient in each defined time interval we obtained the standard deviation of eGFR residuals from the linear regression model used to obtained a eGFR slope [29], and refitted Cox regression models. Results of these sensitivity analyses were very similar and we chose to present only findings of the main analysis.

## Results

Of the 70,598 study patients, 61.2%, 28.9%, and 9.9% were in early stage 3, late stage 3, and stage 4 CKD, respectively. Table 1 also shows that they were largely men (97.5%), and the mean age was 70(±9) years. There were 81.9% white, 14.2% African American, 2.3% Hispanics, and 1.6% were of other racial groups. At the baseline, 36.6% had prior hospitalizations, 69.5% had cardiovascular conditions, 98.3% had other non-cardiovascular conditions, and 19.0% had mental comorbidities. Individuals in stage 4 were more likely to be non-White, and had higher rates of prior hospitalizations and comorbidities than those in stage 3. During follow-up, 19,939 (28.3%) had dialysis-free death, and 7,193 (10.3%) had dialysis. The rate of dialysis or dialysis-free death was higher for patients with more severe CKD: 29.1% for early stage 3, 46.8% for late stage 3, and 73.3% for stage 4 CKD. Overall, the median follow-up time was 3.8 years (range: <0.01-4.8).

**Table 1:** **Death and dialysis outcomes and baseline population characteristics**

	**Total population**	**(N = 70,598; 100%)**	**Early stage 3 CKD**	**(N = 43,228; 61.2%)**	**Late stage 3 CKD**	**(N = 20,387; 28.9%)**	**Stage 4 CKD**	**(N = 6,983; 9.9%)**
	**N**	**%**	**N**	**%**	**N**	**%**	**N**	**%**
**OUTCOMES**								
**Dialysis**	7,193	10.3	1,749	4	2,493	12.4	2,951	42.8
**Dialysis-free death**	19,939	28.3	10,844	25.1	6,957	34.4	2,138	30.5
**CHARACTERISTICS**								
**Age (in years)**								
<45	460	0.6	256	0.6	131	0.7	73	1
45-54	4502	6.4	2663	6.1	1218	6	621	8.9
55-64	11495	16.3	7275	16.9	3005	14.7	1215	17.3
65-74	29068	41.3	19374	45	7123	34.9	2571	37.5
75-84	23993	33.9	13108	30.2	8504	41.8	2381	33.6
85 and above	1080	1.5	552	1.3	406	2	122	1.8
**Sex**								
Men	68818	97.5	42120	97.5	19889	97.6	6809	97.6
Women	1780	2.5	1108	2.5	498	2.4	174	2.4
**Race**								
White	57713	81.9	35886	83.2	16625	81.6	5202	74.6
African American	10017	14.2	5687	13.2	2906	14.3	1424	20.5
Hispanic	1682	2.3	986	2.2	494	2.4	202	2.7
Others	1186	1.6	669	1.4	362	1.7	155	2.2
**VHA priority status**								
Severely disabled	17013	24.1	9835	22.8	5116	24.9	2062	29.8
Moderately disabled	11469	16.2	7100	16.4	3348	16.3	1021	14.7
Poverty	33320	47.2	20749	48	9420	46.3	3151	44.7
Co-pay	7916	11.3	5009	11.6	2237	11.2	670	9.9
Missing	880	1.2	535	1.2	266	1.3	79	0.01
**Prior hospitalizations**								
Yes	25820	36.6	14246	32.9	8276	40.5	3298	47.7
No	44778	63.4	28982	67.1	12111	59.5	3685	52.3
**Cardiovascular conditions**								
Yes	49019	69.5	28666	66.2	15021	0.74	5332	76.9
No	21579	30.5	14562	33.8	5366	0.26	1651	23.1
**Other physical conditions**								
Yes	69391	98.3	42372	98	20104	98.6	6915	99.2
No	1207	1.7	856	2	283	1.4	68	0.8
**Mental comorbidities**								
Yes	13533	19	8467	19.5	3811	18.5	1255	17.8
No	57065	81	34761	80.5	16576	81.5	5728	82.2
**Qualifying eGFR, mean ± sd (in ml/min/1.73 m2)**	46 ± 11		53 ± 4		39 ± 4		24 ± 4	
**Age (in years)**	70 ± 9		70 ± 9		71 ± 9		69 ± 10	

Notes: Because of rounding, percentages may not be equal to 100.

CKD: Chronic Kidney Disease; VHA: Veterans Health Administration; eGFR: estimated Glomerular Filtration Rate; qualifying eGFR is the subsequent eGFR 90 to 365 days apart from the index eGFR; sd: standard deviation.

Generally, eGFR SDs and means decreased as CKD advanced (Table 2). To account for the effect of mean in interpreting SDs as a measure of variability, we have supplemented the statistic of coefficient of variation (defined as standard deviation divided by mean). The summary statistics show that eGFR variability (relative to the mean) increased when CKD progressed. The median coefficient of variation was 10.9 for early stage 3, 12.8 for late stage 3, and 15.4 for stage 4 CKD. The median eGFR SDs from all of the two-year intervals were 5.8, 5.1, and 4.0 ml/min/1.73 m^2^ for early stage 3, late stage 3, and stage 4, respectively. A downward trend of eGFRs was observed for all CKD stages, with the magnitude increasing as CKD progressed. The median slopes went from −2.2 in early stage 3 to −3.5 ml/min/1.73 m^2^/year in stage 4.

**Table 2:** **Summary statistics of eGFRs from a series of two-year moving intervals**

**CKD stage**		**Early 3**						**Late 3**						**Stage 4**					
**Two-year moving interval**																			
		**(−1,0)**	**(0,1)**	**(1,2)**	**(2,3)**	**(3,4)**	**All**	**(−1,0)**	**(0,1)**	**(1,2)**	**(2,3)**	**(3,4)**	**All**	**(−1,0)**	**(0,1)**	**(1,2)**	**(2,3)**	**(3,4)**	**All**
**eGFR**																			
**Variables**																			
**Variability**	**median**	6.0	6.2	5.7	5.3	5.3	5.8	5.5	5.7	4.8	4.4	4.4	5.1	4.6	4.6	3.4	3.1	3.2	4.0
**(SD)**	**Q1**	3.8	4.4	4.1	3.7	3.6	4.0	3.5	3.8	3.4	3.0	2.9	3.4	2.8	3.0	2.4	2.0	2.1	2.6
	**Q3**	8.6	8.6	7.7	7.5	7.6	8.1	8.8	8.4	6.8	6.2	6.3	7.5	7.9	7.1	5.0	4.6	4.8	6.5
	**IQR**	4.8	4.2	3.6	3.8	4.0	4.1	5.3	4.6	3.4	3.2	3.4	4.1	5.1	4.1	2.6	2.6	2.7	3.9
**Mean**	**median**	56.4	55.0	52.8	52	49.9	54.1	43.4	41.7	39.3	38.8	37.6	41.0	29.0	27.1	25.3	25.7	25.8	27.2
	**Q1**	51.9	50.5	47.7	45.8	43.3	48.8	38.5	37.0	34.3	33.0	31.3	35.7	24.6	22.9	20.8	20.9	20.7	22.7
	**Q3**	60.9	59.2	57.6	58	56.7	59.1	48.7	46.5	44.2	44.8	44.2	46.3	34.2	31.4	29.7	31.1	31.8	32.2
	**IQR**	9.0	8.7	9.9	12.2	13.4	10.3	10.2	9.5	9.9	11.8	12.9	10.6	9.6	8.5	8.9	10.2	11.1	9.5
**Coefficient of variation**	**median**	10.7	11.4	10.8	10.4	10.9	10.9	13.1	13.8	12.5	11.5	11.8	12.8	16.3	17.5	14.5	12.4	12.7	15.4
	**Q1**	7.1	8.2	7.7	7.1	7.2	7.5	8.7	9.7	8.8	7.8	8.0	8.7	10.9	12.0	10.1	8.5	8.6	10.5
	**Q3**	14.9	15.6	14.9	14.8	15.7	15.1	18.9	19.4	17.4	16.3	17.2	18.1	24.3	25.1	20.2	17.6	17.8	22.5
	**IQR**	7.8	7.4	7.2	7.7	8.5	7.6	10.2	9.7	8.6	8.5	9.2	9.4	13.4	13.1	10.1	9.1	9.2	12.0
**Slope**	**median**	−3.6	−2.7	−1.3	−1.5	−1.2	−2.2	−5.9	−3.7	−1	−1.5	−1.2	−2.8	−6.8	−4.2	−1.1	−1.4	−1.2	−3.5
	**Q1**	−9.3	−6.9	−5.1	−5.9	−4.6	−6.6	−13	−8.1	−4.4	−5.2	−4	−7.5	−14	−8.3	−3.7	−3.9	−3.2	−8.2
	**Q3**	0.8	0.9	2.7	2.5	2	1.7	−0.8	−0.1	2.7	1.9	1.6	0.8	−2.4	−1.3	1.9	1.2	0.8	−0.3
	**IQR**	10.1	7.8	7.8	8.4	6.6	8.3	12.2	8.0	7.1	7.1	5.6	8.3	11.6	7.0	5.6	5.1	4.0	7.9

CKD: Chronic Kidney Disease; For two-year moving interval, 0 denotes the baseline year, −1 denotes the year prior to the baseline, and 1 to 4 denote the number of years in the follow-up period. eGFR: estimated Glomerular Filtration Rate. SD: standard deviation of an individual patient’s repeatedly measured eGFR values from a two-year interval. Q1: 1st quartile. Q3: 3rd quartile. IQR: Inter-Quartile Range (=Q3 minus Q1). The unit for the eGFR slope variable is ml/min/1.73 m^2^/year.

**Figure 2:**
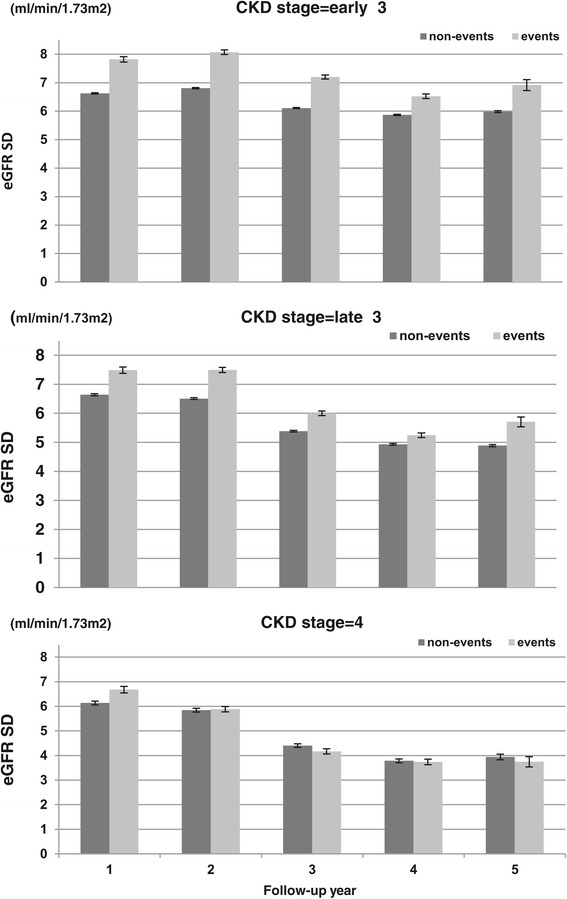
**Average variability (measured by standard deviation) of estimated glomerular filtration rate variability by the follow-up year and presence/absence of events for different chronic kidney disease stages.** CKD – Chronic Kidney Disease. eGFR: estimated Glomerular Filtration Rate .SD: standard deviation. Event: dialysis and/or dialysis-free death. Non-event: absence of dialysis and/or dialysis-free death. The error bar is the standard error for the mean of the estimated Glomerular Filtration Rate variability (measured by standard deviation). Early stage 3 CKD: P < 0.001 for all pairs of the contrast between events vs. non-events. Late stage 3 CKD: P < 0.001 for all pairs of the contrast between events vs. non-events. Stage 4 CKD: P < 0.001 for the first year (p > 0.05 for the other years) of the contrast between events vs. non-events.

Figure 2 delineates the relationship between variability in eGFRs (SDs) and presence/absence of the events of dialysis or dialysis-free death. Across all three CKD stages and for all follow-up years, individuals with events always had significantly (p < 0.001 from t-tests) greater average SDs than those without events, with the exception of the last four follow-up years in stage 4 (p > 0.05).

In Table 3, results from Cox regression models show that a greater eGFR SD was associated with elevated risk of dialysis or dialysis-free death, with or without adjusting for other independent variables. For example, in early stage 3, 1 ml/min/1.73 m^2^ increase in eGFR SD in prior two years would increase the risk of dialysis or dialysis-free death in the current year by 7.6% after adjusting for the eGFR means and slopes (Model 1, adjusted hazard ratio (AHR) = 1.076; 95% CI = (1.071, 1.080)), or by 7.2% after additionally adjusting for other demographic and clinical variables (Model 2, AHR = 1.072; 95% CI = (1.067, 1.080)). Similar associations were found in the other CKD stages. Interactions of eGFR SDs and slopes were not statistically significant.

**Table 3:** **Adjusted hazard ratios for dialysis or dialysis-free death, by CKD stage**

**CKD stage**	**Early stage 3**						**Late stage 3**						**Stage 4**					
	**Model 1**			**Model 2**			**Model 1**			**Model 2**			**Model 1**			**Model 2**		
	**AHR**	**95% CI**		**AHR**	**95% CI**		**AHR**	**95% CI**		**AHR**	**95% CI**		**AHR**	**95% CI**		**AHR**	**95% CI**	
		**Low**	**High**		**Low**	**High**		**Low**	**High**		**Low**	**High**		**Low**	**High**		**Low**	**High**
**Variables**																		
**eGFR variability (SD)**	1.076	1.071	1.080	1.072	1.067	1.076	1.080	1.074	1.085	1.076	1.069	1.082	1.076	1.063	1.089	1.073	1.061	1.085
**eGFR mean**	0.963	0.961	0.966	0.968	0.965	0.970	0.950	0.948	0.953	0.951	0.948	0.954	0.928	0.922	0.933	0.927	0.922	0.933
**eGFR slope (ml/min/1.73 m2 per year; 10 unit decrease)**	1.011	1.004	1.018	1.010	1.003	1.017	1.007	1.004	1.009	1.008	1.006	1.011	1.064	1.035	1.094	1.067	1.038	1.096
**Age (in years)**																		
45-54				0.83	0.63	1.08				0.93	0.73	1.19				0.91	0.66	1.25
55-64				0.89	0.68	1.16				0.93	0.73	1.19				0.86	0.63	1.18
65-74				1.17	0.90	1.51				0.98	0.77	1.24				0.85	0.62	1.15
75-84				1.58	1.22	2.05				1.15	0.91	1.46				0.82	0.60	1.12
85 and above				2.71	2.05	3.57				1.78	1.37	2.31				1.07	0.75	1.52
<45 (reference)				1						1						1		
**Sex**																		
Women				0.63	0.54	0.72				0.64	0.55	0.76				0.66	0.54	0.81
Men (reference)				1						1						1		
**Race/ethnicity**																		
African American				1.07	1.02	1.13				1.10	1.03	1.16				1.14	1.06	1.22
Hispanic				0.96	0.85	1.09				0.91	0.79	1.05				0.93	0.79	1.10
Others				0.82	0.70	0.96				0.95	0.81	1.11				1.05	0.87	1.27
White (reference)				1						1						1		
**VHA priority status**																		
Severely disabled				1.26	1.21	1.32				1.17	1.11	1.23				1.2	1.12	1.28
Moderately disabled				0.96	0.91	1.01				0.93	0.87	0.98				1.03	0.95	1.12
Co-pay required				0.79	0.74	0.84				0.86	0.8	0.93				0.92	0.83	1.02
Poverty (reference)				1						1						1		
**Prior hospitalization**				1.56	1.50	1.62				1.48	1.42	1.55				1.28	1.21	1.37
**Cardiovascular conditions**				1.52	1.45	1.59				1.38	1.31	1.46				1.21	1.13	1.30
**Mental comorbidities**				1.12	1.07	1.17				1.10	1.04	1.16				1.06	0.99	1.14

CKD: Chronic Kidney Disease; Model 1: Containing eGFR variation (SD), mean, and slope variables; Model 2: Model 1 plus all other independent variables (age, sex, race/ethnicity, VHA priority status, presence of hospitalization, cardiovascular conditions, and mental comorbidities); AHR: Adjusted Hazard Ratio; CI: Confidence Interval; All variables except for eGFR SD, mean and slope variables (time-varying variables) were derived from the baseline period. The indicator of presence of non-cardiovascular conditions (Table 1) was not included in the modeling because about 99% of patients had the conditions; eGFR: estimated Glomerular Filtration Rate; SD: standard deviation; VHA: Veterans Health Administration. The 880 people in the missing category of VHA priority status (Table 1) was not included for this analysis; We rounded the estimates for eGFR SD, mean and slope to the third decimal place to allow differentiation between the AHR, and low and high limit of the 95% CIs.

Other independent variables were also significantly related to the risk of dialysis or dialysis-free death. If the eGFR declined by 10 ml/min/1.73 m^2^ per year in previous two years, the risk would increase by 1.0% (Model 2, AHR = 1.010; 95% CI = (1.003, 1.017)) in early stage 3 CKD, and 6.7% (Model 2, AHR = 1.067; 95% CI = (1.038, 1.096) in stage 4 CKD. Compared to patients younger than 45 years old, patients 65 or older in early stage 3, 75 or older in late stage 3, and 85 or older in CKD 4 were at greater risk. Women tended to have lower risk (AHR range: 0.63 to 0.66) than men. African Americans, but not other racial/ethnic minorities were at higher risk (1.07 to 1.14) than Whites. Individuals with severe service connected disability also had higher risk (1.17 to 1.26) compared to others. As regards clinical factors, individuals with hospitalization history (1.28 to 1.56), presence of cardiovascular conditions (1.21 to 1.52) and mental comorbidities (early stage 3: 1.12; late stage 3: 1.10) at the baseline had elevated risks. The magnitude of the AHR for these clinical factors somehow decreased when CKD advanced.

## Discussion

In this large cohort study of longitudinal outpatient eGFR values, we found that greater variability in eGFRs, measured by SDs in a dynamic/time-varying fashion over the follow-up, was associated with elevated risk of dialysis or dialysis-free death, even after controlling for downward trends (slopes) in CKD progression and other demographic and clinical factors. In all three CKD stages, one ml/min/1.73 m^2^ increase in a patient’s SD of eGFRs from prior two years was significantly associated with about 7% increase in risk of dialysis/death in current year (e.g., adjusted hazard ratio in early stage 3:1.072; 95% CI = (1.067, 1.080)). A recent study [19] of stage 3-5 CKD patients also found the variability in repeatedly measured eGFRs to be associated with death, although eGFR variability was measured differently and only death was evaluated as the outcome in the study. Both studies used VHA patients and have detailed individual-level electronic health records in the VHA health care system. However, our study had Medicare information for totality of data rather than VHA data alone (most VHA patients are also Medicare eligible). Additionally, we simultaneously evaluated eGFR slopes and eGFR variability as potential risk factors by including both as time-varying independent variables in the statistical models. We found that a faster downward eGFR progression was associated with increased risk of CKD outcomes. This is consistent with the literature [7] and the common knowledge among clinicians caring for CKD patients.

Since our findings support the hypothesis of eGFR variability (measured by standard deviation) being associated with increased risk of dialysis or dialysis free death, the key message for clinicians or health policy makers is to consider using a simple statistic—standard deviation, to assist in clinical care of CKD patients. We have proposed to measure eGFR variability using the statistic of standard deviation because it reflects the essence of the concept and is familiar among clinicians and policy makers. The derivation of this statistic can be easily built into any existing systems of electronic health records and the result (value) can be instantly made available. Because this statistic is a summary measure of the variability (non-stability) of renal function, it can be used as a tool by clinicians in assessing CKD management for a patient and assist in clinical decision making for CKD care. For example, a clinician can compare a patient’s current statistic with earlier results or the norm (to be established as future work) and an elevated value (indicating increased variability of renal function) may alert the clinician to an acute kidney injury or other reasons for such increased variability. This might direct clinicians to look into the patient’s history of eGFR values and health conditions, and to engage in active dialogues with the patient regarding the patient’s recent changes of health conditions and/or medications that may affect kidney function. Standard deviation decreases as mean decreases (also as seen in Table 2). Since the renal function of patients with CKD is expected to decline over time (hence decreased eGFR values), one would expect their standard deviations of eGFR values decrease as well. Therefore, it is important to note that in comparing a patient’s past and current eGFR SDs, an increased SD would not be expected and may warrant more detailed assessment.

Although variability in eGFR in CKD progression may have not been well studied, the concept of variability has been applied to different physiological markers in medicine and is supported by various biological mechanisms. For example, within-visit variability of blood pressure was associated with stroke and all-cause mortality [30]. Heart rate variability predicted worst outcomes in perioperative and intensive care settings [31]. Furthermore, home blood pressure and heart rate variability were associated with cardiovascular events [32], and hemoglobin (Hb) variability was associated with higher mortality among patients with ESRD [29] or type 2 diabetes [33]. In patients with type 1 diabetes, HbA1C variability was predictive of development of microalbuminuria, progression of kidney disease and cardiovascular events [34].

In this study, we propose to measure eGFR variability as a proxy for kidney resilience to stimuli. Greater variability of eGFR values over time means decreased renal function and more stable eGFR values over time means less damaging renal function. We caution not to confuse this with the renal reserve (RR) test. The renal reserve measures ability of a kidney to increase basal GFR after a protein overload [35]; a larger value means a better kidney ability/function. In CKD patients, renal reserve is usually preserved but with a lower magnitude, suggesting that remaining nephrons are able to maintain some response to protein overload despite already being in a state of hyperfiltration [35,36].

Understanding predictors and pathways to ESRD/dialysis in patients with CKD is important. A recent study reported that patients were highly concerned with the uncertainty of CKD progression and that physicians were frustrated with their inability to predict the course of CKD [37]. Uncertainty in predicting CKD progression may limit clinicians’ ability in effective CKD management and impair preparation for dialysis such as vascular access placement for high-risk patients [38]. Understanding CKD progression and identifying patients at high risk of adverse outcomes is also valuable for health care resource and program planning to improve CKD care. The uncertainty rises mainly due to acute changes, eGFR variability, and/or other characteristics of the course of CKD progression, already stated above. Pathways other than linear slopes have been described, but require a rather difficult application of statistical modeling [12]. Prospective clinical studies may help to address these issues. We suggest that regional health maintenance organizations with electronic health records attempt to replicate our findings. We also recognize that the general unavailability of electronic medical records makes implementation of eGFR variability problematic. However, the evaluation of individual level longitudinal data using electronic records are consistent with Federal policies to establish meaningful use criteria; it provides the opportunity to simultaneously improve quality, safety, and efficiency and the results can provide decision support for national high-priority conditions.

We would like to note that there are existing studies addressing the question of changes in repeatedly measured eGFR values. It was reported that patients in the quartile with the greatest eGFR change were more likely to incur death [39-41] and ESRD in one study [42] although not in another [43]. In these studies, eGFR change was defined as annual decline (measured as change in eGFR values from two visits relative to the time elapsed between these visits) [39] or percentage of change in eGFR values (measured as change in eGFR values from two visits relative to the first eGFR value) [42] or both [40], or mean absolute eGFR residual values [41,43]; patients then were assigned into quartiles based on the value of eGFR change. The findings of these studies were largely consistent with ours, although different approaches were used.

There are a few unique features of this study: 1) the advanced modeling of variability, slopes, and means of repeated eGFR values over time as time-dependent variables (calculated in each two-year interval with sensitivity analyses) allows us to effectively test the hypothesis and address the research question; 2) The explicit adjustment of eGFR slopes (as a time-dependent covariate) allows us to evaluate whether there is an interaction between eGFR variability and CKD progression or only existence of their main effects; 3) inclusion of both dialysis and death as adverse CKD outcomes for evaluation provides us overall understanding of risks of two critical adverse outcomes of CKD progression in relation to eGFR variability; and 4) evaluation of the associations by CKD stage permits us to compare whether findings depend on baseline renal status.

Our study has limitations. First, the findings of the study may not be generalized beyond populations of largely male, elderly and with diabetes; and CKD patients identified based on two eGFR values less than 60 ml/min/1.73 m^2^. Second, we were not able to identify transient or temporary dialysis, resulting in censoring some patients whose kidney function was resilient and their creatinine values later improved during the follow-up. Lastly, the primary goal of this study was to evaluate eGFR variability as a new risk factor in CKD progression in order to enhance clinical decisions by clinicians, and resources planning by policy makers/administrators; nonetheless, assessing factors affecting eGFR variability as future work is warranted. We suspect variability in eGFRs may be related to medication changes, acute diseases (infections, cardiac events, etc.) and hospitalizations (although we included only outpatient creatinine values in this study). Indeed, in an effort to assess eGFR variability as a risk factor without the influence of some severe cardiac conditions, we conducted two separate additional analyses where we excluded 1) patients with a prior history of CHF at baseline or 2) patients with CHF anytime during the study period (but prior to CKD outcomes if applicable). The findings remained similar.

## Conclusions

In conclusion, this study provides support for evaluating eGFR variability as an independent risk factor of dialysis/death, regardless of CKD stage. It has important clinical implication because patients may benefit from monitoring of eGFR variability in addition to their rates of eGFR decline.

## Additional file

Additional file 1:
**Codes used in the study for determination of dialysis and access (fistula procedure).** Description: This file lists codes used in the study for determination of dialysis in the baseline period (to remove study subjects) and in the outcome years (for study of dialysis as an outcome).

## Abbreviations

CKD: Chronic kidney disease
eGFR: Estimated glomerular filtration rate
VHA: Veterans health administration
HMO: Health maintenance organization
SD: Standard deviation
ESRD: End-stage renal disease
CHF: Congestive heart failure
FY: Fiscal year
AHR: Adjusted hazard ratio
CI: Confidence interval
