# Supplementary material for: Variability in estimated glomerular filtration rate values is a risk factor in chronic kidney disease progression among patients with diabetes
Source: BMC Nephrol. 2015 Mar 25;16:34. doi: 10.1186/s12882-015-0025-5 (PMC4377072; doi:10.1186/s12882-015-0025-5)
Supplement: Additional file 1: — Codes used in the study for determination of dialysis and access (fistula procedure). Description: This file lists codes used in the study for determination of dialysis in the baseline period (to remove study subjects) and in the outcome years (for study of dialysis as an outcome). [file 12882_2015_25_MOESM1_ESM.docx]

**Additional File 1: Codes used in the study for determination of dialysis and access (fistula procedure)**

**The baseline period:**

To determine if a veteran should be excluded in the baseline period defined as within the 1 year period prior to the qualifying date of the eGFR, we used a broad definition of all dialysis and access related codes. This consisted of International Classification of Diseases, Ninth Revision, Clinical Modification (ICD-9-CM) diagnosis and procedure codes, Current Procedural Terminology (CPT) codes, revenue codes (Medicare Part A), and Veterans Health Administration (VHA) clinic stop codes. The complete listing for dialysis related codes is as follows:

ICD-9-CM diagnostic codes (V56, V56.x, 458.21, E879.1, V45.1),

ICD-9-CM procedure codes (3895, 3995, 5498),

CPT codes (90921, 90925, 90935, 90937, 90945, 90947, 90999, 49421),

Medicare Part A revenue codes (0801, 0802, 0803, 0804, 0809, 0821, 0831, 0841, 0851, 0881), and

VHA stop codes (602, 603, 604, 606, 607, 608, 610, 611).

For access (fistula procedure) related codes: ICD-9-CM procedure codes (3927, 3942, 3943), and

CPT codes (36821, 36825, 36830, 36831, 36833, 49422, 93990).

**The follow-up period:**

In the follow-up time period to determine the outcome variable of time to first dialysis, codes explicitly referring to dialysis treatment were used:

ICD-9-CM diagnostic codes (V56, V56.8, V45.1),

ICD-9-CM procedure codes (3995, 5498),

CPT codes (90935, 90937, 90945, 90947, 90999),

Medicare Part A revenue codes (0801, 0802, 0803, 0804, 0821, 0831, 0841, 0851), and

VHA stop codes (602, 603, 606, 607).
